# Relationships between body image, nutritional supplement use, and attitudes towards doping in sport among adolescent boys: implications for prevention programs

**DOI:** 10.1186/1550-2783-11-13

**Published:** 2014-03-27

**Authors:** Zali Yager, Jennifer A O’Dea

**Affiliations:** 1 College of Education, Victoria University, Melbourne, Australia; 2 Faculty of Education and Social Work, University of Sydney, Sydney, Australia

**Keywords:** Body image, Performance-enhancing drugs, Image-enhancing drugs, Nutritional supplements, Doping, Male adolescent

## Abstract

**Background:**

Reports of high levels of use of protein powders and nutritional supplements among young men is a concern because these substances may act as a gateway for the use of drugs and illegal substances to enhance appearance or sports performance. The aim of this study was to investigate the relationship between body dissatisfaction, weight change behaviors, supplement use, and attitudes towards doping in sport among an adolescent male sample.

**Methods:**

Participants were 1148 male adolescents (age range 11-21 years) in Australia who completed a self-report questionnaire that measured weight change behaviors, supplement use, body dissatisfaction (Male Body Attitudes Scale; MBAS) and attitudes towards doping in sport (Performance Enhancing Attitudes Survey; PEAS).

**Results:**

There was a positive correlation between MBAS total and PEAS scores (r = .19, p < .001), indicating that the young men who were more dissatisfied with their bodies were more likely to support the use of doping in sport. Young men who were currently attempting weight loss or weight gain, and those currently consuming energy drinks (**η**p^2^ = .01, p < .01) and vitamin/mineral supplements (**η**p^2^ = .01, p < .01) were also significantly more supportive of doping in sport. However, those involved in weight lifting, and using protein powders were not (p > .05).

**Conclusions:**

These findings suggest that body dissatisfaction, weight change behaviors, and supplement use are related to more lenient attitudes towards doping in sport among adolescent boys. Future research might examine whether combining educational content for the prevention of body dissatisfaction and the use of drugs in sport may have a greater preventive impact than current programs aimed at young men.

## Background

The most recent decade of research has revealed that body image is an issue for young men [1,2]. Around two-thirds of adolescent boys are dissatisfied with their bodies, and this is equally split between those who desire weight loss and those who want to gain muscle [3-6]. The most recent prevalence study, conducted in Greece, indicated that the levels of body dissatisfaction were similar for adolescent boys and girls [1]. An early Australian study reported that 59.4% of adolescent boys were ‘currently trying to build up their body’ and 74% believed that they should ‘develop their muscles’ [7]. Interviews with adolescent boys revealed that half wanted to change their body weight, and the majority of participants thought that body size, shape and muscularity was important [8]. Body dissatisfaction is known to lead to the adoption of deleterious weight change strategies such as excessive exercise, and the use of supplements, some of which may be drugs and/or substances that are banned in sports competitions [9]. Cross-sectional and longitudinal research has confirmed that body dissatisfaction and drive for muscularity predict the consumption of nutritional supplements such as protein powders, as well as anabolic steroids [10-12].

The consumption of these substances is a concern due to the immediate harms of use, but also the potential that they will eventually act as a gateway to more serious substances and illegal drug use. Gateway theory proposes that adolescents who use legal substances such as alcohol and cigarettes are more likely to progress to using more and more serious, illegal drugs, in a predictable pattern [13]. There is some recent evidence to suggest that the same progression also occurs for performance and image enhancing supplements, in that the use of protein powders and supplements predicts the use of anabolic steroids [14,15]. In a study of 212 male and female competitive adolescent athletes, those who were taking nutritional supplements were 3.5 times more likely to report doping, and had significantly more positive attitudes towards the use of drugs in sport [14]. Users of anabolic-adrenergic steroids [AAS] have also been found to be more likely to engage in the consumption of alcohol and other drugs [16,17].

Doping, as defined by the World Anti-Doping Agency, is any violation of the rules set out in the Anti-Doping Code, which range from possession, use, and trafficking of illegal substances to engaging in behaviors associated with illegal substances, and refusing, or tampering with sample collection for drug testing (WADA, 2009). These behaviors are also known as the use of performance enhancing drugs, and ergogenic aids. These actions are strongly governed among the athlete community, however there is sound evidence that doping behaviors are becoming more prevalent among the general population [18,19]. Male non-athletes have been found to have the highest usage levels of doping and ergogenic aids, followed by recreational athletes - while elite athletes report the lowest rates of use [20]. Males in the general population report using these substances to gain muscle in order to increase strength, improve their physical performance, and enhance their appearance [18]. In addition, the main reasons given for desired weight gain among male adolescents are to increase physical strength, protect themselves, and improve sports performance [7]. Young men want to succeed in physical endeavors regardless of their level of competition. The difference is that the use of supplements among elite athletes is heavily regulated; however in recreational settings, those who use these supplements are not penalized, and use may therefore occur freely, regardless of the impacts on health [18].

The Internet has increased accessibility to information about, and sales of, weight gain supplements. Young males are aware of the products and substances that are available via the Internet for performance and image enhancement [18]. However, knowledge of negative side effects of supplement use is generally poor, as evidenced by one study, in which none of the adolescents could name any potential risks or negative side effects of the supplements and nutritional products that they were consuming [21]. Research from the USA reported that 14.7% of young males in grades 8–12 stated that they would take a pill or potion that was guaranteed to help them meet their fitness goals, even if it would harm their health and 8.6% indicated that they would do so even if it shortened their life [22]. Nilsson and colleagues (2005) also found that those Swedish young men who had taken steroids were less likely to believe that these drugs were harmful [16]. Laboratory studies have demonstrated that at least one in five protein supplements is contaminated [23], and the American Academy of Pediatrics (2005) reports that the harm of performance and image enhancing drug use by adolescents is relatively unexplored and unknown [24]. Side effects of supplement and doping use in adults have also not been fully explored, but can include psychiatric symptoms, aggression, renal failure, increased blood pressure, cardiovascular disease and death [25].

Previous research has focused on investigating the prevalence of supplement use and the relationship between doping and body dissatisfaction among elite athletes [14,26] and university students [15]. However, no prior studies have explored the relationship between body dissatisfaction, use of weight gain supplements, and attitudes towards performance enhancing drugs in sport among adolescent boys. This research aimed to examine the relationship between body dissatisfaction and attitudes towards drug use in sport in order to inform education and health promotion programs.

We hypothesized that:

– 1) adolescent boys with higher levels of body dissatisfaction will be more likely to report using products and supplements for weight gain;

– 2) adolescents who use nutritional products and are engaged in weight lifting will be more likely to endorse the use of performance enhancing drugs in sport; and that

– 3) adolescent boys with increased body dissatisfaction will have positive attitudes towards drug use in sport.

## Methods

Data collection took place as a part of a 12-year cross-sectional follow –up of early work by the second author [7]. Following institutional (University of Sydney) and departmental (NSW Department of Education) ethics approval, schools were recruited through contact with the principal. All males enrolled in school years 7–12 in each of the consenting schools were invited to participate in the study during their regular class time. Boys gave their signed consent to participate in the study, as did their parents. The questionnaire was introduced and explained to the students by the researchers, who supervised the session to ensure that the boys completed their own work in silence. Participant’s height and weight were measured and recorded by the researchers using calibrated digital scales and a portable stadiometer. In total, data were collected from 1148 male students in 28 public, private and catholic schools in NSW, Australia.

The questionnaire used in this study was based on the initial instrumentation that was used in the year 2000 [27], with additional items and standardized scales [28,29] added to collect a wider range of data and reflect the changing nature of measurement and methodologies in public health. The questionnaire was not changed significantly enough to warrant pilot testing. Table 1 provides the details of the measures used in this study.

**Table 1:** Measures used to assess body image, supplement use, and attitudes towards doping in sport

**Variable**	**Measure**
Body image	Direct questioning: (*Do you think you are: Too fat/ just right/ too thin*) and (“*Would you like to be*: *A little lighter, A lot lighter, The same as it is now, Heavier, A lot heavier*”) [6]
Current attempts to gain or lose weight	Direct questioning ‘*Are you currently trying to gain weight’ (Yes/No)* and ‘*Are you currently trying to lose weight’ (Yes/No)*[6].
Sports participation	Direct questioning about participation in weight lifting asked participants to indicate their levels of sports involvement [*Not involved, Recreational, Serious, Weight lifting or Body building*]
Supplement use	Direct questioning about the use of sports drinks, vitamins and minerals, energy drinks, protein powders, herbal supplements, and creatine in the past two weeks (*yes/no*)
Body dissatisfaction	Male Body Attitudes Scale [MBAS] [28]. Participants responded on a likert-type scale [*Never = 1*, to *Always = 6*] includes three subscales of dissatisfaction with height (2 items), fatness (12 items), muscle (14 items), and a total score (24 items). Chronbach’s Alpha was .92.
Attitudes towards performance enhancing drugs	Performance Enhancement Attitudes Scale [PEAS] [29]. Participants respond to 17-items on a Likert scale [Strongly disagree = 1, to Strongly agree = 6]. Chronbach’s Alpha was .89.

Quantitative data were entered into SPSS and cleaned. Height and weight were used to calculate Body Mass Index [BMI] (weight divided by height squared), and weight status was determined using BMI cutoffs appropriate for each age group [30]. Standardized measures of body image [MBAS] and attitudes towards doping [PEAS] were scored in accordance with the instructions of those who designed the measures and mean scores were generated. Higher scores on these measures indicate higher levels of body dissatisfaction [MBAS], and more lenient attitudes towards doping in sport [PEAS]. Significant Kolmogov-Smirnov statistics indicated that total scores on subscales and final scales for these measures were not normally distributed. Transforming these scores using square root did not restore normality, so non-parametric versions of tests were used wherever possible. ANOVA and t-tests were used to determine the difference between the adolescent boys’ use of weight gain strategies, supplements and attitudes towards doping by age. These were checked with Kruskal-Wallace tests, the nonparametric alternative. There is no non-parametric alternative for ANCOVA, which was used to determine the difference in mean scores on MBAS and PEAS scales by other participant characteristics, controlling for age. We used Pearson’s correlations to determine the strength and direction of the relationship between MBAS and PEAS scores. Effect sizes (partial eta squared) are also reported. Descriptive statistics were used to determine results for the items using direct questioning. Open-ended written responses were analyzed by determining categories and tallying responses in order to present frequencies of these comments.

## Results

### Demographics

A total of *N* = 1148 young men completed the survey. Age ranged from 11.08-21.08 years and the mean age was 14.56 [SD = 1.48]. The majority of participants reported their ethnic/cultural background as Caucasian/ Northern European (53.3%, *n* = 610). Other cultural backgrounds included Southern European (25.1%, *n* = 287), South East Asian/Chinese (10%, *n* = 114), Middle Eastern/Arabic (3.8%, *n* = 44), Aboriginal/ Torres Straight Islander (2.9%, *n* = 33), Polynesian/Maori (2.5%, *n* = 29), Indian or Sri Lankan (1.5%, *n* = 17) and African (1%, *n* = 11). The Socio-Economic Status [SES] of each school was ranked as low/medium/high using the Australian national government school data [31]. Participants were distributed by SES as follows: low SES (37.3%, *n* = 428); middle SES 35.9% ( *n* = 412) and high SES 26.8% (*n* = 307).

The majority of participants (68.3%, *n* = 784) were classified as being within the healthy weight range, according to their BMI. A further 19.5% (*n* = 224) of participants were considered to be overweight, 7.7% (*n* = 88) were classified as obese, and 4.5% (*n* = 52) were underweight.

### Body image

In response to the direct questions about body image and body dissatisfaction, 77.7% (*n* = 883) of adolescent boys indicated that they would describe their body as ‘about right’, while 9.8% (*n* = 111) selected ‘too thin’ and 12.5% (*n* = 142) selected ‘too fat’. In contrast, for the question about body dissatisfaction, only 35.2% (*n* = 400) of adolescent boys indicated that they would like to be their present weight, with 30.0% (*n* = 341) indicating that they would like to be a little lighter, and 27.6% (*n* = 314) indicated that they wanted to be a little heavier.

Previous research indicates that levels of body image and dissatisfaction vary according to age. We therefore analyzed the differences in MBAS subscale scores according to participants’ age category. These data are presented in Table 2.

**Table 2:** Male Body Attitudes Survey [MBAS] scores by age

	**12 year olds and under (*n*** **= 176)**	**13 year olds (*n*** **= 262)**	**14 year olds (*n*** **= 286)**	**15 year olds (*n*** **= 215)**	**16 year olds and over (*n*** **= 205)**	**Total group (*n*** **= 1148 )**	**ANOVA**
	**Mean [ *SD *]**	**Mean [ *SD *]**	**Mean [ *SD *]**	**Mean [ *SD *]**	**Mean [ *SD *]**	**Mean [ *SD *]**	
MBAS height (Score out of 12)	6.25 [3.05]	6.41 [2.83]	6.27 [2.78]	5.91 [2.67]	6.49 [2.78]	6.26 [2.82]	F(4,1101) = 1.32
MBAS fat (score out of 48)	19.72 [8.75]	19.34 [8.28]	19.56 [8.31]	19.12 [8.42]	19.18 [8.77]	19.40 [8.45]	F(5,1002) = 0.64
MBAS muscle (score out of 60)	25.81 [9.85]	26.72 [9.91]	27.17 [9.70]	28.37 [9.20]	31.30 [10.55]	27.64 [9.91]	F(4, 993) = 9.79***
MBAS total score (score out of 144)	62.61 [22.18]	61.52 [20.01]	62.67 [20.50]	64.17 [20.27]	67.06 [21.82]	63.31 [20.84]	F(4, 888) = 3.79**

Note: **p < 0.01; ***p < 0.001.

Total MBAS scores, and dissatisfaction with muscularity increased with age. Males older than 16 years of age were significantly more likely to have higher scores on the muscularity subscale and the total MBAS indicating greater levels of body dissatisfaction. Dissatisfaction with height and fatness were relatively stable across age groups. In contrast, there was no significant difference in mean PEAS scores by age group. The mean (SD) total score on the PEAS was 38.25 [13.93] for the whole cohort.

### Body image by use of products

We asked participants whether they had consumed certain nutritional products or supplements in the past 2 weeks. Almost half (49.9%) of the participants had consumed sports drinks such as Gatorade and Powerade, and 37.0% had taken vitamins or minerals. Almost one –third of the boys had consumed ‘energy drinks’ such as Red Bull, and a quarter had used protein powder such as Sustagen (24.8%). The following table (Table 3) provides the MBAS scores of those who had, or had not recently used these products, after controlling for the effect of age.

**Table 3:** Male Body Attitudes Scale [MBAS] subscale scores of adolescent boys using certain products and supplements in the past two weeks

	**Used**	**Not used**	**ANCOVA**^**a**^	**Effect size**	**Effect of age**
	**Mean [ *SD *]**	**Mean [ *SD *]**		**ηp**^**2**^	
Vitamins and Minerals					
• MBAS height	6.37 [2.85]	6.24 [2.82]	F(1,1100) = 0.60	.001	NS
• MBAS fatness	19.82 [9.01]	19.52 [8.36]	F(1,1001) = 0.41	.000	NS
• MBAS muscularity	31.34 [11.25]	29.92 [10.69]	F(1,992) = 5.37*	.005	***
• MBAS total score	65.12 [22.17]	63.31 [20.34]	F(1,887) = 1.90	.002	***
Protein powder					
• MBAS height	6.68 [2.74]	6.15 [2.85]	F(1,1100) = 6.77*	.006	NS
• MBAS fatness	21.09 [9.20]	19.16 [8.35]	F(1,1001) = 8.63**	.009	NS
• MBAS muscularity	33.41 [10.99]	29.48 [10.72]	F(1,992) = 20.94***	.02	***
• MBAS total score	69.89 [21.85]	62.04 [20.40]	F(1,887) = 20.89***	.02	***
Sports drinks (eg., Gatorade, Powerade)					
• MBAS height	6.32 [2.80]	6.25 [2.86]	F(1,1100) = .18	.000	NS
• MBAS fatness	19.55 [8.51]	19.71 [8.70]	F(1,1001) = .08	.000	NS
• MBAS muscularity	31.79 [10.72]	29.08 [10.94]	F(1,992) = 16.44***	.02	***
• MBAS total score	65.19 [20.59]	62.62 [21.40]	F(1,887) = 3.30	.00	***
Energy drinks (e.g., Red Bull, V, Mother)					
• MBAS height	6.44 [2.66]	6.22 [2.90]	F(1,1100) = 1.28	.00	NS
• MBAS fatness	20.26 [8.92]	19.36 [8.45]	F(1,1001) = 1.39	.00	NS
• MBAS muscularity	33.38 [10.76]	29.16 [10.74]	F(1,992) = 22.07***	.02	***
• MBAS total score	68.06 [20.18]	62.17 [21.16]	F(1,887) = 10.26**	.01	**

Note: *p < 0.05; **p < 0.01; ***p < 0.001 ^a^ANCOVA controlled for age in these analyses.

Hypothesis one was supported. Adolescent boys who had taken vitamins and minerals, protein powder, sports drinks and energy drinks were significantly more likely to be dissatisfied with their muscularity. Those participants who had used protein powders in the last two weeks had significantly higher scores on all of the MBAS subscales, indicating that they had higher levels of body dissatisfaction. Age was significant in some, but not all, of these analyses, as shown in Table 3.

In the open-ended questions, we asked participants to indicate why they had consumed these products. Most (37.3%, n = 426) indicated that they had used them to improve their sports performance, while only 4.8% (n = 55) indicated that they used them to gain weight. Many young men (38.6%, n = 441) indicated that they had used them for other reasons, which included ‘wanting to’ or ‘liking it’, ‘needing energy’, and consuming them for ‘taste’. Others indicated that they consumed them for ‘energy, health, to recover from illness’, or ‘because their doctor told them to’.

### Relationship between doping attitudes, body dissatisfaction and weight change behaviours

The following table (Table 4) provides the PEAS scores of men who had, or had not engaged in a range of weight change strategies.

**Table 4:** Relationship between doping attitudes [PEAS scores], weight change, weightlifting, and nutritional product use in the past two weeks

	**Yes**	**No**	**ANCOVA**^**a**^	**Effect size**
	**Mean [ *SD *]**	**Mean [ *SD *]**		**ηp**^**2**^
Current weight gain	42.95 [15.38]	37.67 [13.93]	F(1,669) = 11.82**	.02
Energy drinks	41.09 [16.03]	37.36 [13.19]	F(1,672) = 9.81**	.01
Vitamins and minerals	40.46 [15.21]	37.47 [13.54]	F(1,672) = 6.87**	.01
Current weight loss	40.81 [13.16]	37.69 [14.41]	F(1,667) = 5.93*	.009
Sports drinks	39.50 [13.86]	37.48 [14.41]	F(1,672) = 3.42	.005
Protein powder	40.33 [15.51]	37.94 [13.75]	F(1,672) = 3.19	.005
Weightlifting	41.09 [17.90]	38.17 [14.76]	F(1,673) = 2.49	.004

Note: *p < 0.05; **p < 0.01; ^a^ANCOVA controlled for age in these analyses.

Hypothesis two was partially supported. Adolescent boys who indicated that they were currently trying to lose weight and currently trying to gain weight were significantly more likely to have higher scores on the PEAS, indicating more lenient attitudes towards doping in sport. Participants who indicated that they had taken vitamins and minerals, and consumed energy drinks in the past two weeks were significantly more likely to have higher scores on the PEAS measures, indicating that they were more supportive of doping in sport. However, there was no significant difference in the PEAS scores of young men who had recently used protein powder and sports drinks, or were involved in weight lifting. The effect of age was not significant in any of the ANCOVA analyses.

### Relationship between body dissatisfaction and attitudes towards doping in sport

We utilized Pearson’s correlations to examine the relationship between participants’ body dissatisfaction and attitudes towards doping in sport. Table 5 presents these data.

**Table 5:** Pearson’s correlations between measures of body dissatisfaction and attitudes towards doping in sport

	**MBAS height**	**MBAS fatness**	**MBAS muscularity**	**MBAS total score**	**PEAS total score**
MBAS height	-	.19***	.30***	.42***	.04
MBAS fatness		-	.53***	.84***	.16***
MBAS muscularity			-	.88***	.20***
MBAS total score				-	.19***
PEAS total score					-

***p < .0001 (2-tailed).

There were significant correlations between participants’ scores on MBAS subscales. There were also significant, positive relationships between PEAS total scores, total MBAS scores, and MBAS subscales of dissatisfaction with fat and muscularity. This indicates that those participants who had higher levels of body dissatisfaction were more likely to support doping in sport, indicating that hypothesis three was supported. Although these correlations were significant, the strength of the correlation was still small according to Cohen’s guidelines [small = .10-.29; medium = .30-.49; large = .50-1.0] [32].

## Discussion

This research provides evidence of a significant relationship between body image, use of weight change products, and attitudes towards the use of doping. We found that the adolescent boys who reported using supplements and dietary products such as vitamins and minerals, protein powders, sports drinks and energy drinks were more likely to have higher levels of body dissatisfaction. Those participants who had used vitamins and minerals, and energy drinks (but not those who had used sports drinks and protein powders) were also more likely to have more lenient attitudes towards the use of drugs in sport. Furthermore, this research is the first study to report a significant, positive correlation that indicates that adolescent boys who have higher levels of body dissatisfaction are also more likely to be more supportive of the use of drugs in sport.

Our findings reflect those of previous work in this area. Male adolescents who reported using steroids have been found to have poorer body image [33] and to engage in disordered eating behaviors [11]. Recent work also reported significantly more positive attitudes towards doping among older adolescent male competitive athletes who reported using nutritional supplements [14]. Our findings add evidence of positive attitudes towards doping in sport among a universal sample of adolescent males who are attempting weight change, dissatisfied with their body (and in particular, their muscularity) and using products such as vitamins and minerals, and energy drinks.

This relationship has a wide range of implications for education, health promotion and drug prevention programs. School-based programs aiming to reduce body dissatisfaction among adolescent boys have not been widely successful [34]. This may be because the nature of body image prevention programming that focuses on discussions about the influence of peers and the media, and was designed for young girls, is not particularly appealing for adolescent boys. While young girls are primarily interested in becoming thinner for aesthetic reasons [35] males are interested in the functional aspects of their body [36], and state that they take supplements, and engage in weight change behaviors for the purposes of gaining strength and improving sports performance [7]. Therefore, body image prevention programming that focuses on body acceptance ignores one of the primary drivers for men engaging in weight change behaviors.

Similarly, interventions to prevent the use of steroids among adolescents have not targeted body dissatisfaction. Nilsson and colleagues recognized the necessity of targeting appearance in interventions to reduce steroid use, and trialed a program in Sweden, but did not use standardized measures in their evaluation [37].

The Athletes Training and Learning to Avoid Steroids [ATLAS] program [38] represents exemplary practice in the prevention of anabolic steroid use among high school athletes in the USA. This program has undergone two decades of systematic testing with extensive follow-up periods. The most recent trial involved 3207 male adolescent football players in the USA, who participated in a program that included nutrition information, role plays of drug refusal, analysis of media influences, and discussion of alternate strategies to improve performance. This theory-based session was supported by a weights session in the gym each week. Classes were conducted by coaches and trainers, and achieved significant increases in self esteem, knowledge about, attitudes towards, and intentions to use anabolic steroids [38]. Further research could investigate whether there are any long term outcomes from such interventions and whether future drug use attitudes or behaviors are associated with educational initiatives that are delivered consistently from a young age.

It is suggested that programs aiming to prevent the development of body dissatisfaction and the use of performance and image-enhancing drugs could be combined due to the potential to target shared risk factors. It is likeley that content focusing on alternatives to the use of performance and image-enhancing products and doping could make body image programming more relevant for young males. Materials could include information about the potential harmful effects of using protein supplements and other image and performance-enhancing drugs in the same way that we discuss the dangers of dieting for girls. Programs could then provide information about safe and effective ways to achieve increases in strength and sports performance without the use of products and doping. Similarly, the inclusion of evidence-based body image materials, such as media literacy, and cognitive dissonance activities, in doping prevention programs could enhance the impact of these interventions.

One final element of a combined intervention program could revolve around the theme of fair play. Some researchers suggest that doping prevention needs to go beyond health education, to examine the moral and ethical basis for decision making in relation to doping in sport [39], and report that trials comparing the effects of a decision-making intervention with traditional psycho-educational approaches are underway [39]. WADA’s prevention and education program ‘*Play True Generation’* includes a range of materials aimed at engaging young athletes with the moral issues surrounding doping in sport [40]. The *Play True Challenge*, an online game where players are able to practice making decisions about doping in sport, and see the consequences of these actions, could easily be incorporated into prevention programming, and the high school health and physical education curriculum.

This research offers new insight into the relationships between body image, nutritional product use, and attitudes towards doping in sport. Strengths included the large sample, and the use of standardized measures to assess body image and attitudes towards doping in sport. However, there were also some limitations. Very few adolescent men reported using performance enhancing drugs, and we don’t know if this was due to the lack of comfort they felt in reporting this information. In addition, adolescent men might have misinterpreted the meaning of some of the words and concepts in this questionnaire, which might have resulted in inaccurate reporting. We suggest that future research tracks these relationships longitudinally in order to determine whether body dissatisfaction predicts the use of drugs in sport.

## Conclusions

This research suggests that there is a relationship between body image, use of supplements, and attitudes towards the use of drugs in sport. These findings suggest that health educators, sports professionals and administrators could take a different preventive approach that combines the prevention of doping with complementary interventions to prevent body dissatisfaction. Universal and targeted prevention that takes this approach for males is likely to improve the physical and psychological health of young boys in the short term, as they are less likely to suffer from body dissatisfaction, and the negative side effects of using supplements and anabolic steroids. Widespread dissemination of this approach to prevention could then have flow-on effects for reducing doping behaviors in competitive sports.

## Competing interests

The authors declare that they have no competing interests.

## Authors’ contributions

J O’D and ZY obtained grant funding for this project. J O’D conducted data collection and instructed research assistants in data entry. ZY prepared data files and conducted data analysis. ZY and J O’D prepared this manuscript for publication. Both authors read and approved the final manuscript.
